# Severity of underweight and risk of fracture: a Korean nationwide population-based cohort study

**DOI:** 10.1038/s41598-022-14267-x

**Published:** 2022-06-16

**Authors:** Sangsoo Han, Jiwon Park, Sangun Nah, Hae-Dong Jang, Kyungdo Han, Jae-Young Hong

**Affiliations:** 1 Department of Emergency Medicine, Soonchunhyang University Bucheon Hospital, 170 Jomaru-ro, Bucheon, 14584 Republic of Korea; 2 Department of Orthopedics, Korea University Hospital, Ansan, 123, Jeokgeum-ro, Danwon-gu, Ansan-si, Gyeonggi-do 15355 Republic of Korea; 3 Department of Orthopaedic Surgery, Soonchunhyang University Bucheon Hospital, 170 Jomaru-ro, Bucheon, 14584 Republic of Korea; 4 Department of Statistics and Actuarial Science, Soongsil University, 369 Sangdo-ro, Dongjak-gu, Seoul, 06978 Republic of Korea

**Keywords:** Health care, Risk factors

## Abstract

Underweight is an important modifiable risk factor for fractures. However, there have been few large cohort studies regarding the relationship between underweight and fracture in the general population. We investigated the risk of fracture development according to underweight severity in a large population cohort. This nationwide cohort study included 2,896,320 people aged ≥ 40 years who underwent national health checkups in 2009 and were followed up to identify the incidence of fracture until December 31, 2018. After applying the exclusion criteria that included overweight and obese individuals, the study population was divided according to body mass index (BMI) into normal weight (18.5 ≤ BMI < 23.0), mild underweight (17.5 ≤ BMI < 18.5), moderate underweight (16.5 ≤ BMI < 17.5), and severe underweight (BMI < 16.5) groups. Cox proportional hazards regression analyses were performed to calculate the hazard ratios for risk of fracture according to underweight severity. Severely underweight participants had a 28% increased fracture risk (adjusted hazard ratio [HR] 1.28, 95% confidence interval [CI] 1.20–1.37) compared with those of normal weight. In addition, fracture risk was increased by 14% in individuals with moderate underweight (adjusted HR 1.14, 95% CI 1.08–1.19) and 9% in those with mild underweight (adjusted HR 1.09, 95% CI 1.06–1.13). The severity of underweight was significantly associated with risk of fracture.

## Introduction

Fractures are one of the leading health problems in adults and one of the leading causes of morbidity. Fractures are also closely associated with social costs because they can lead to prolonged absence from work, use of significant medical resources, and long-term disability^1^. Well-known risk factors associated with fractures include old age, female sex, low body mass index (BMI), current smoking, excessive drinking, and physical activity^2,3^. Among these, underweight is an important modifiable risk factor because it is associated with osteoporosis or sarcopenia^4,5^.

Bodyweight is an important determinant of health status, including metabolic, immune, reproductive, and musculoskeletal systems^6^. People with underweight may have a poor physical condition, which is strongly associated with increased morbidity and mortality^7,8^. In addition, being underweight may increase the risk of fractures because it may be associated with low bone density, soft tissue loss, and muscle weakness^5^. To date, many studies on the relationship between fractures and BMI have classified underweight simply as BMI < 18.5 kg/m^2^^9,10^. However, many people (especially women) control their weight more excessively than do those of normal weight due to negative attitudes and discrimination against overweight people in modern society^11^. Therefore, a study using a more detailed classification of underweight is needed in consideration of these aspects. The World Health Organization classifies underweight into categories as mild, moderate, and severe underweight^12^.

Therefore, we evaluated the risk of fracture according to underweight severity using a community-based database in South Korea, which includes > 500,000 individuals participating in health examinations. Furthermore, we adjusted for as many known fracture-related risk factors as possible in a large-scale population-based database.

## Methods

### Study design and population

This nationwide population-based study was performed using the Korean National Health Insurance Service (NHIS) database. NHIS is a compulsory national health insurance system that covers approximately 99% of the population of the Republic of Korea for almost all medical processes. NHIS provides regular health check-ups to adults over 40 years old and workers over 20 years old every 1–2 years and obtains information on anthropometric measurements, alcohol consumption, smoking status, and medical history via self-reported questionnaires and laboratory findings. Self-reported data were double-checked through an interview with a doctor, and blood sampling was performed after fasting for at least 8 h. The NHIS claims database contains extensive health information for more than 50,000,000 Koreans. This database includes data regarding diagnoses and comorbidities coded according to the International Classification of Diseases, Tenth Revision (ICD-10), demographic characteristics, prescription medications, health care services such as treatments, and costs for outpatients and inpatients^13^. Since 2015, the NHIS has provided access to this Korea-representative retrospective cohort database to all researchers whose study protocols have been approved by an official review committee. All data are anonymized, collected regularly, and subjected to careful quality control. This NHIS database has been used in many epidemiological studies in various fields, and its validity has been confirmed in previous studies^14,15^.

From the NHIS database, approximately 4 million participants were randomly selected using simple random sampling. We included individuals over 40 years old who underwent health examinations by the NHIS in 2009. Subjects were excluded if baseline characteristic data were missing, they had a prior diagnosis of fracture before enrollment or a fracture occurring during the 1-year lag period, or they were overweight or obese (BMI ≥ 23.0).

### Key variables

#### Classification of underweight

BMI was calculated by dividing body weight (kilograms) by height squared (m^2^). The study population was divided into four groups: normal weight (18.5 ≤ BMI < 23.0), mild underweight (17.5 ≤ BMI < 18.5), moderate underweight (16.5 ≤ BMI < 17.5), and severe underweight (BMI < 16.5)^12^.

#### Incidence of fracture

To confirm the causes of all fracture cases, we used ICD-10 codes and hospitalization records from the NHIS system. Fractures were defined using the ICD-10 codes as follows: vertebral fractures (S12.0, S12.1, S12.2, S22.0, S22.1, S32.0, M48.4, and M48.5), hip fractures (S72.0, S72.1, and S72.2), and other fractures, including upper arm fractures (S42.0, S42.2, and S42.3), forearm fractures (S52.5 and S52.6), and lower leg fractures (S82.3, S82.5, and S82.6). A fracture was defined as one hospitalization and/or ≥ two outpatient visits within 1 year with the relevant ICD-10 codes. From January 1, 2010 to December 31, 2018, fracture cases were confirmed by checking the NHIS medical claim records. Participants who died during the follow-up period were censored at the time of death.

#### Covariates

The NHIS database includes data on demographics, socioeconomic status, comorbidities, and laboratory findings, such as total cholesterol level, blood glucose level, and estimated glomerular filtration rate. Participants were classified according to smoking status as non-smokers, ex-smokers, or current smokers. Participants were classified according to alcohol consumption as non-drinkers, moderate drinkers (< 30 g/day), or heavy drinkers (≥ 30 g/day)^16^. Regular exercise was defined as at least 20 min of high-intensity physical activity ≥ 3 days per week or at least 30 min of moderate-intensity physical activity ≥ 5 days per week^17^. Low income was defined as an income < 20th percentile.

Diabetes was defined as a fasting blood sugar level > 126 mg/dL or prescription of an antidiabetic agent (ICD-10 codes, E11–E14). Hypertension was defined as an average blood pressure ≥ 140/90 mmHg, or more than one annual prescription of an antihypertensive agent (ICD-10 codes, I10–I13 or I15). Dyslipidemia was defined as a total cholesterol level ≥ 240 mg/dL or more than one annual prescription of an antihyperlipidemic agent (ICD-10 code, E78). Chronic kidney disease (CKD) was defined as an estimated glomerular filtration rate < 60 mL/min/1.73 m^2^. Previous studies validated the definitions of comorbidities based on the ICD codes^15,18^.

### Statistical analysis

Statistical analyses were performed using the chi-squared test for categorical variables and analysis of variance (ANOVA) for continuous variables. The incidence rate (IR) was calculated by dividing the outcome rate per 1000 person-years (PY) by the total number of fractures. The 95% confidence intervals (CIs) and hazard ratios (HRs) for fractures based on underweight severity were calculated using Cox regression analysis. We constructed a hierarchical model with different levels of demographic, socioeconomic factors, and comorbidities to investigate covariates potentially affecting fracture risk: model 1 was non-adjusted; model 2 was adjusted for age and sex; model 3 was additionally adjusted for other factors, including alcohol consumption, smoking status, low income, and regular exercise; and model 4 was fully adjusted with additional adjustments for comorbidities such as diabetes, hypertension, dyslipidemia, and CKD. We also compared the cumulative incidences of fractures between groups using the Kaplan–Meier method. To examine the effects of clinical conditions on the association between risk of fracture and underweight severity, the HRs for fractures in diverse subgroups were determined by Cox proportional hazards regression analysis and *P* values for interaction. Stratified subgroup analysis was performed based on age (< 65 and ≥ 65 years old), sex, smoking status, alcohol consumption, household income, regular activity, and comorbidities. All statistical analyses were performed using SAS software (ver. 9.3; SAS Institute, Cary, NC, USA). A two-sided *P* < 0.05 was taken to indicate statistical significance.

### Ethics statement

The study protocol was approved by the NHIS institutional review board. The informed consent requirement was waived because the data analyses were performed retrospectively using anonymous data derived from the NHIS database in Korea. This study was also approved by the institutional review board of Korea University Ansan Hospital, Republic of Korea (approval no. K2021-2601-001). All research processes were conducted in accordance with the appropriate regulations and guidelines, and this study was performed in accordance with the provisions of the Declaration of Helsinki.

## Results

**Figure 1:**
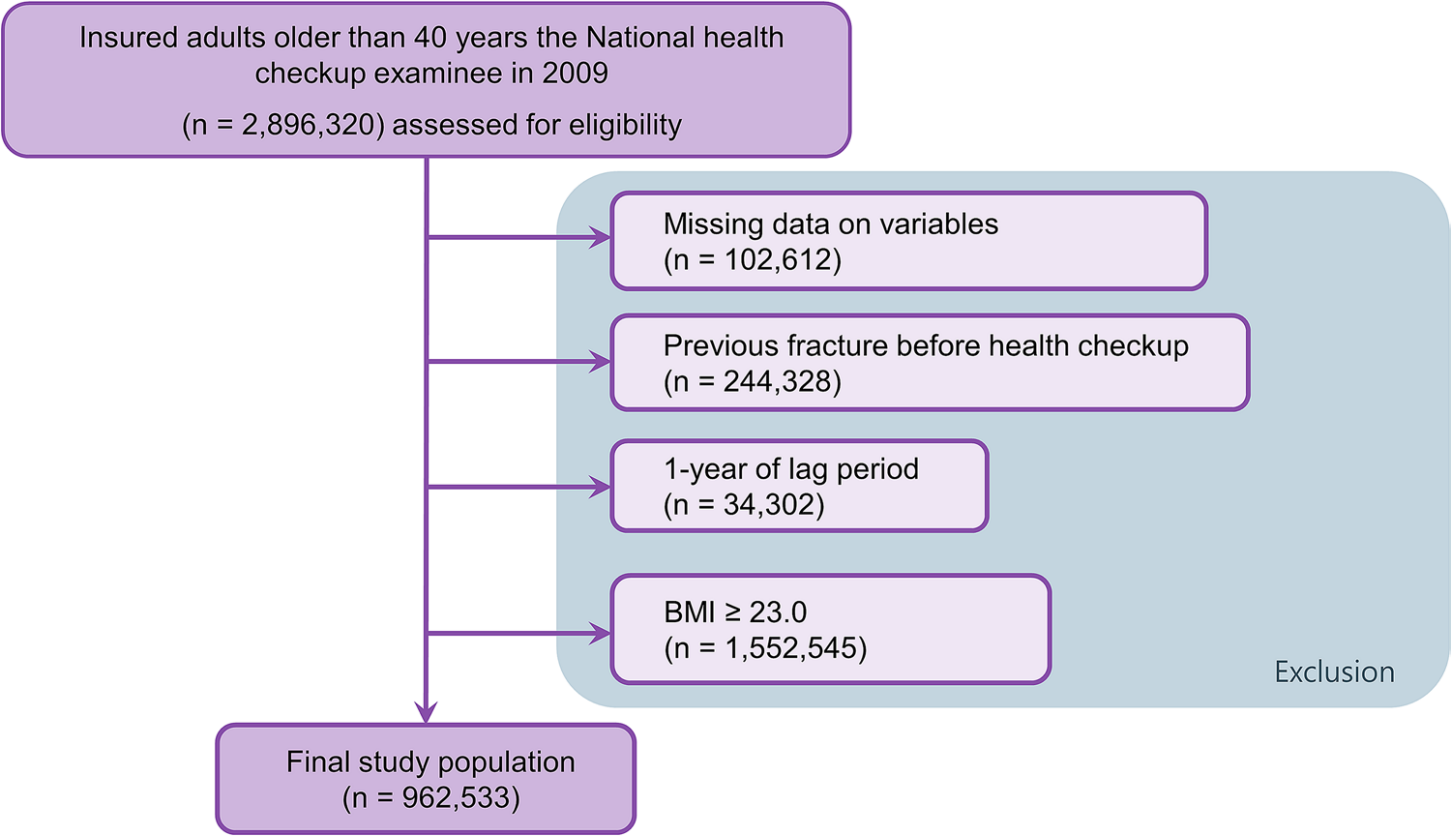
Flow chart of the study population. *BMI* body mass index.

A total of 2,896,320 adults over the age of 40 years underwent NHIS-provided health check-ups in Korea in 2009. A total of 102,612 individuals were excluded due to missing data on variables, 244,328 were excluded due to previous fractures before study enrollment, and 34,302 were excluded due to a fracture occurring during the 1-year lag period. In addition, 1,552,545 overweight or obese individuals were also excluded. Finally, 962,533 subjects were included in the analysis (Fig. 1).

### Baseline characteristics

A descriptive overview of the participants’ characteristics according to underweight severity is presented in Table 1. A total of 5695 (0.59%) participants were classified as belonging to the severely underweight group consisting of 2619 men (45.99%) and 3076 women (54.01%) with a mean age of 60.72 years. The moderate underweight group included 13,071 (1.36%) participants consisting of 6393 men (48.91%) and 6678 women (51.09%) with a mean age of 56.62 years. The mild underweight group included 36,283 (3.77%) participants consisting of 16390 (45.17%) men and 19,893 women (54.83%) with a mean age of 54.36 years. Finally, the normal weight group comprised 907,484 (94.28%) participants consisting of 406,439 men (44.79%) and 501,045 women (55.21%) with a mean age of 53.06 years.

**Table 1:** Baseline characteristics of participants according to the degree of underweight.

	Severe underweight (*n* = 5695)	Moderate underweight (*n* = 13,071)	Mild underweight (*n* = 36,283)	Normal weight (*n* = 907,484)	*P* value
	BMI < 16.5	16.5 ≤ BMI < 17.5	17.5 ≤ BMI < 18.5	18.5 ≤ BMI < 23.0	
**Demographics**					
Male sex, *n* (%)	2619 (45.99)	6393 (48.91)	16,390 (45.17)	406,439 (44.79)	< 0.0001
Age, years	60.72 ± 13.74	56.62 ± 13.16	54.36 ± 12.22	53.06 ± 10.45	< 0.0001
≥ 65 years	3237 (56.84)	9085 (69.51)	27,818 (76.67)	759,791 (83.73)	< 0.0001
BMI, kg/m^2^	15.72 ± 0.8	17.06 ± 0.27	18.03 ± 0.29	21.29 ± 1.16	< 0.0001
*Smoking status, n (%)*					< 0.0001
Non-smoker	3591 (63.06)	8019 (61.35)	2362 (63.56)	602,237 (66.36)	
Ex-smoker	531 (9.32)	1249 (9.56)	3593 (9.9)	112,965 (12.45)	
Current smoker	1573 (27.62)	3803 (29.09)	9628 (26.54)	192,282 (21.19)	
*Alcohol consumption, n (%)*					< 0.0001
Non-drinker	4099 (71.98)	8698 (66.54)	23,486 (64.73)	550,731 (60.69)	
Mild drinker	1283 (22.53)	3651 (27.93)	10,811 (29.8)	303 503 (33.44)	
Heavy drinker	313 (5.5)	722 (5.52)	1986 (5.47)	53,250 (5.87)	
Regular exercise, *n* (%)	655 (11.5)	1784 (13.65)	5101 (14.06)	172,189 (18.97)	< 0.0001
Low income, *n* (%)	1174 (20.61)	2557 (19.56)	7076 (19.5)	167,385 (18.44)	< 0.0001
**Comorbidities**					
Hypertension, *n* (%)	1423 (24.99)	2685 (20.54)	6781 (18.69)	216,730 (23.88)	< 0.0001
Diabetes, *n* (%)	500 (8.78)	925 (7.08)	2238 (6.17)	7143 (7.83)	< 0.0001
Dyslipidemia, *n* (%)	609 (10.69)	1288 (9.85)	3663 (10.1)	146,740 (16.17)	< 0.0001
CKD, *n* (%)	571 (10.03)	960 (7.34)	2498 (6.88)	63,163 (6.96)	< 0.0001
**Fracture**					
Incidence, *n* (%)	222 (3.9)	294 (2.25)	554 (1.53)	6647 (0.73)	< 0.0001

*BMI* body mass index, *CKD* chronic kidney disease.

The incidence of fracture was highest in the severe underweight group (222, 3.9%), followed by the moderate underweight group (294, 2.25%), mild underweight group (554, 1.53%), and normal weight group (6647, 0.73%).

### Severity of underweight and fracture

**Figure 2:**
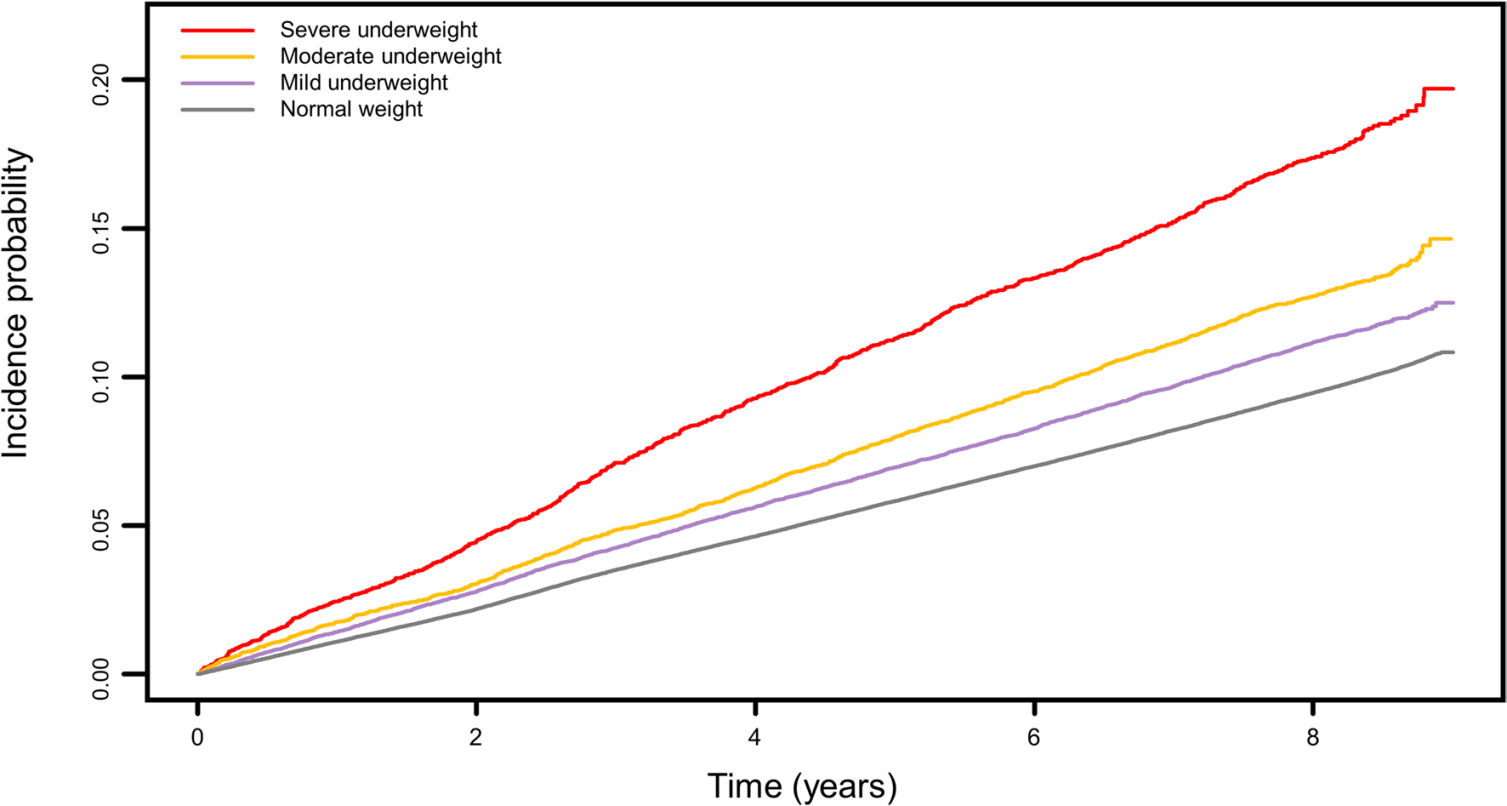
Kaplan–Meier estimates of fracture incidence according to underweight severity.

Cox proportional hazards regression analyses were performed to determine the risk of fracture in participants according to underweight severity (Table 2). The normal weight group served as the reference for fracture events. Participants with severe underweight showed markedly higher risks of subsequent fracture, despite adjustments for several potential confounders (adjusted HR 1.28, 95% CI 1.20–1.37). The Kaplan–Meier curves for each group are shown in Fig. 2, and the results showed a significantly higher fracture incidence in the severe underweight group than in the other groups at all time points (log-rank test, *P* < 0.001).

**Table 2:** Calculated hazard ratios for fracture according to the degree of underweight.

Group	Events	FU duration (PY)	IR (per 1000 PYs)	Hazard ratio (95% confidence interval)			
				Model 1	Model 2	Model 3	Model 4
Severe underweight	908	37,833.2	24.0	1.93 (1.81–2.06)	1.30 (1.21–1.38)	1.27 (1.19–1.36)	1.28 (1.20–1.37)
Moderate underweight	1616	94,971.9	17.0	1.36 (1.30–1.43)	1.14 (1.09–1.20)	1.13 (1.07–1.19)	1.14 (1.08–1.19)
Mild underweight	4025	272,731.5	14.8	1.18 (1.14–1.22)	1.10 (1.06–1.13)	1.09 (1.05–1.12)	1.09 (1.06–1.13)
Normal weight	88,434	7,066,609.0	12.5	1.00 (reference)	1.00 (reference)	1.00 (reference)	1.00 (reference)

Model 1: non-adjusted. Model 2: adjusted for age and sex. Model 3: adjusted for age, sex, smoking status, alcohol consumption, low income, and regular exercise. Model 4: Adjusted for age, sex, smoking status, alcohol consumption, low income, regular exercise, diabetes, hypertension, dyslipidemia, and chronic kidney disease.

*FU* follow-up, *IR* incidence rate, *PY* person-year(s).

### Subgroup analysis

Subgroup analyses were performed by stratifying the study population according to age, sex, smoking status, alcohol consumption, low income, and comorbidities. The adjusted fracture risks according to underweight severity in each subgroup are presented in Table 3. The association between underweight severity and fracture risk was similar across the subgroups. In the subgroups of age > 65 years, male sex, hypertension, and diabetes, severe underweight had a greater effect on fracture risk. No significant differences were observed in the other subgroup analyses of fracture risk according to smoking status, alcohol consumption, low income, dyslipidemia, and CKD (P > 0.05).

**Table 3:** Fracture risk subgroup analyses according to the degree of underweight.

Category	Hazard ratio (95% confidence interval)				*P* for interaction
	Severe underweight	Moderate underweight	Mild underweight	Normal weight	
**Age**					
≥ 65 years	1.41 (1.27–1.56)	1.08 (1.00–1.16)	1.06 (1.01–1.10)	1	< 0.0001
< 65 years	1.22 (1.12–1.33)	1.21 (1.13–1.29)	1.16 (1.11–1.22)	1	
**Sex**					
Male	1.64 (1.47–1.82)	1.31 (1.22–1.41)	1.24 (1.18–1.31)	1	< 0.0001
Female	1.11 (1.02–1.21)	1.03 (0.97–1.10)	1.01 (0.97–1.06)	1	
**Hypertension**					
No	1.27 (1.17–1.37)	1.11 (1.05–1.18)	1.06 (1.02–1.10)	1	< 0.0001
Yes	1.29 (1.15–1.45)	1.19 (1.09–1.31)	1.18 (1.12–1.26)	1	
**Diabetes**					
No	1.27 (1.18–1.36)	1.13 (1.07–1.19)	1.08 (1.04–1.11)	1	< 0.0001
Yes	1.39 (1.13–1.72)	1.22 (1.03–1.43)	1.28 (1.16–1.42)	1	

Adjusted for age, sex, smoking status, drinking status, low income, regular exercise, diabetes, hypertension, dyslipidemia, and chronic kidney disease. There were no significant differences in other subgroup analyses, which included associations of fracture risk with smoking, drinking, regular exercise, low income, dyslipidemia, and chronic kidney disease.

## Discussion

The present study was performed to investigate the association between underweight severity and the development of fracture in a nationwide general population in Korea. Underweight was subdivided into mild (17.5 ≤ BMI < 18.5), moderate (16.5 ≤ BMI < 17.5), and severe underweight (BMI < 16.5). The risk of fracture increased in proportion to underweight severity, with severely underweight individuals showing the highest fracture risk in the general population. To our knowledge, this is the first study to examine the association between underweight severity and fracture risk using real-world large population-based data.

Similar to our findings, previous studies reported that underweight is associated with fracture risk^19,20^ This association may be explained by the following hypotheses. First, lower body weight is associated with less soft tissue, including around the bones. As subcutaneous fat can act as a buffer against damage, it is advantageous for maintenance of bone structure and strength^21^. Second, underweight is associated with low bone mineral density. This association can be explained by the gravitational effect of body weight on bones, along with the effects of body fat and lean body mass^6,22^. Third, underweight may be associated with deficiencies in nutrients, such as vitamin D and protein^23^. Low vitamin D levels has been shown to be associated with defective mineralization of collagenous matrix (osteoid)^24^, and protein depletion may affect the bone remodeling process by reducing the production of insulin-like growth factor 1^25^. Fourth, Gariballa et al. reported a significantly increased prevalence of sarcopenia in underweight patients compared with those with normal or increased body weight^4^. Decreased muscle mass may not provide adequate bone protection, and reduced muscle strength may increase the risk of fall-related injuries^26,27^.

In the present study, older underweight subjects (≥ 65 years) showed a greater fracture risk compared with younger subjects (< 65 years). A previous study showed that underweight in the elderly was associated with malnutrition and osteoporosis, both of which are risk factors for fracture^23^. Comorbidities may be associated with the occurrence of fractures, and in the present study, participants with high blood pressure or diabetes also had a higher fracture risk. Hypertension can cause long-term impairment of calcium homeostasis, resulting in persistent calcium loss in the urine, which increases the rate of mineral loss^28^. In addition, high blood pressure is associated with a high level of parathyroid hormone, which can accelerate bone turnover and decrease bone mass^29^. Metabolic effects of diabetes, such as acidosis and hypercalcemia, may increase the risk of fractures^30^.

Our results may have important implications from a clinical and public health perspective, supporting the need to avoid severe underweight in adults over 40 years of age and to avoid becoming severely underweight to reduce the risk of fractures. Although our data could not show that the risk of fractures is reduced when severe underweight is improved, our results can help clinicians explain to patients that severe underweight may increase their risk of fractures.

A notable strength of this study is that our results can be generalized to the Korean population because we used large-scale population-based data representing the whole country to analyze the relationship between underweight severity and fracture risk. However, this study had some limitations. First, our findings could not determine causal relationships due to its retrospective design. In addition, we attempted to adjust for possible confounding factors, but it is possible that some confounders remained. Second, we could not take bone mineral density into consideration because of a lack of relevant information in the NHIS database. Third, we could not determine the cause of the fracture. In the NIHS database, there was insufficient information on injury mechanisms or laboratory findings to determine the cause of fractures. Further well-designed prospective studies are needed to overcome these limitations.

## Conclusion

Underweight severity was shown to be associated with fracture risk in the general Korean population. Individuals who were more severely underweight had a higher risk of fractures. In particular, the risk of fracture was significantly higher in men than in women, individuals ≥ 65 years than in those < 65 years, and subjects with hypertension and diabetes than in those without these comorbidities.
